# Academic Stress, Subsequent Physical Activity, and Depressive and Anxiety Symptoms Among College Students: A Three-Wave Longitudinal Study

**DOI:** 10.3390/bs16091629

**Published:** 2026-09-11

**Authors:** Haoxuan Ruan, Qiuhan Zhu

**Affiliations:** 1 Research Center for Sports Humanities and Social Sciences, Tianjin University of Sport, Tianjin 300381, China; 2 School of Kinesiology and Physical Education, Zhengzhou University, Zhengzhou 450001, China

**Keywords:** academic stress, physical activity, exercise intention, depressive symptoms, anxiety symptoms, college students, longitudinal study, indirect association

## Abstract

College students’ physical activity may decline when academic demands are high, even among those who previously intended to exercise. This three-wave longitudinal study examined whether T2 physical activity, modeled conditional on T1 exercise intention and T1 physical activity, was involved in a time-ordered indirect association between T1 academic stress and T3 depressive and anxiety symptoms. Of 982 students who completed the baseline survey in September 2025, 750 provided complete matched data in December 2025 and March 2026 (retention = 76.4%). Primary models used raw T2 physical activity and adjusted all component, direct, and total-effect equations for the identical covariate set: gender, age, BMI, T1 exercise intention, T1 physical activity, T1 PHQ-9, and T1 GAD-7. A sign-reversed residualized activity score was examined only as an algebraically equivalent parameterization. T1 exercise intention predicted higher T2 physical activity (*β* = 0.308), whereas T1 academic stress was associated with lower T2 physical activity (B = −12.771, *p* < 0.001). Under identical adjustment, raw T2 physical activity was inversely associated with T3 depressive symptoms (B = −0.0024) and anxiety symptoms (B = −0.0013); the corresponding sign-reversed residualized coefficients were equal in magnitude and opposite in sign, with identical R^2^ values (0.4257 and 0.2720). Full-process bootstrap indirect associations were significant for depressive symptoms (B = 0.0309, 95% CI [0.0229, 0.0396]) and anxiety symptoms (B = 0.0162, 95% CI [0.0099, 0.0236]). The Stress × Intention interaction and quadratic residualized-activity terms were not significant. No observed T1 variable differed significantly between retained and attrited participants. Higher academic stress was associated with relatively lower subsequent physical activity, which was in turn statistically associated with later depressive and anxiety symptoms. The findings do not establish causality, disruption of intention enactment, or a distinct psychological residual mechanism.

## 1. Introduction

Depressive and anxiety symptoms are common during the university years, a period characterized by academic evaluation, identity development, changing social networks, financial demands, and increasing autonomy (Auerbach et al., 2018; Li et al., 2022). Their relevance extends beyond symptom prevalence: internalizing symptoms can interfere with academic engagement, sleep, social functioning, quality of life, and help-seeking, while academic conditions themselves can intensify psychological strain (Barbayannis et al., 2022; Campbell et al., 2022). A useful next step is to identify proximal, modifiable behavioral processes that are longitudinally associated with both academic stress and later symptoms.

Physical activity is one plausible behavioral process (Huang et al., 2025; Yue et al., 2026; W. Zhang et al., 2025). International guidance identifies regular activity as a core component of health promotion (Bull et al., 2020), and prospective meta-analyses associate higher activity with lower subsequent risk of depression and anxiety (McDowell et al., 2019; Pearce et al., 2022). These associations do not establish that activity causes symptom change, particularly when activity and symptoms are self-reported. Nevertheless, physical activity remains a relevant campus-health target because it is modifiable and may support affect regulation, mastery, social contact, sleep, and recovery from study demands.

The theory of planned behavior positions intention as a proximal antecedent of action (Ajzen, 1991, 2020), and a large body of literature documents that intention does not always translate into behavior (Feil et al., 2023; Rhodes & Dickau, 2012; Rhodes & de Bruijn, 2013; Sheeran, 2002; Sheeran & Webb, 2016; Webb & Sheeran, 2006). The present study, however, does not directly measure a conventional intention–behavior gap. Such a measure would require intended and enacted behavior to be defined for the same action, unit, and time period. Here, T1 intention referred to regular exercise over the following three months, whereas the T2 IPAQ-SF assessed total physical activity during the previous seven days and included walking and other activity that may not correspond to intended exercise. Intention is therefore used as a prior behavioral predictor and covariate rather than as evidence that a participant failed to enact a specific target.

Post-intentional theories nevertheless provide a cautious rationale for examining subsequent activity conditional on prior intention. Academic stress may be associated with lower later activity because workload, fatigue, rumination, and schedule disruption reduce discretionary time and opportunities for exercise (Bélanger-Gravel et al., 2013; Kwasnicka et al., 2016; Schwarzer, 2008; Sniehotta et al., 2005). A stronger claim that stress specifically weakens intention enactment would require both better matched intention–behavior measures and evidence that stress changes the intention–activity slope. The present study therefore distinguishes an additive association between stress and activity from moderation of the intention–activity relationship.

The primary intermediate variable was raw T2 physical activity. T1 exercise intention and T1 physical activity were included as covariates, so the T2 activity coefficient was interpreted conditionally. For transparency, we also calculated a sign-reversed residualized T2 activity score: positive values indicate activity below the level predicted by T1 intention and T1 activity. This residual is a statistical reparameterization of T2 activity, not an independently measured psychological construct or a participant-specific target shortfall. Under identical covariates, raw-activity and residualized models necessarily have identical fitted values and R^2^.

Lower physical activity may be associated with later symptoms through reduced opportunities for affect regulation, mastery, social interaction, sleep support, and recovery (McDowell et al., 2019; Pearce et al., 2022). Self-discrepancy or perceived failure is another possible explanation, but the present study did not assess goal attainment, remembered intention, frustration, competence, or perceived control (Higgins, 1987). Those accounts remain hypotheses for future research rather than mechanisms tested here.

Few studies have temporally separated academic stress, subsequent physical activity, and later depressive and anxiety symptoms while simultaneously controlling prior activity, prior exercise intention, and both baseline symptom measures. The contribution of this study is therefore not the proposal of a new residual construct; it is the three-wave evaluation of a conditional physical-activity pathway, accompanied by an explicit comparison showing that raw and residualized parameterizations contain the same linear predictive information.

Five hypotheses were evaluated and are summarized in Table 1.

**H1.**  *Stronger T1 exercise intention is associated with higher T2 physical activity.*

**H2.**  *Higher T1 academic stress is associated with lower T2 physical activity.*

**H3.**  *Higher T2 physical activity is associated with lower T3 depressive symptoms.*

**H4.**  *Higher T2 physical activity is associated with lower T3 anxiety symptoms.*

**H5.**  *T2 physical activity statistically accounts for time-ordered indirect associations between T1 academic stress and each T3 symptom outcome.*

**Figure 1:**
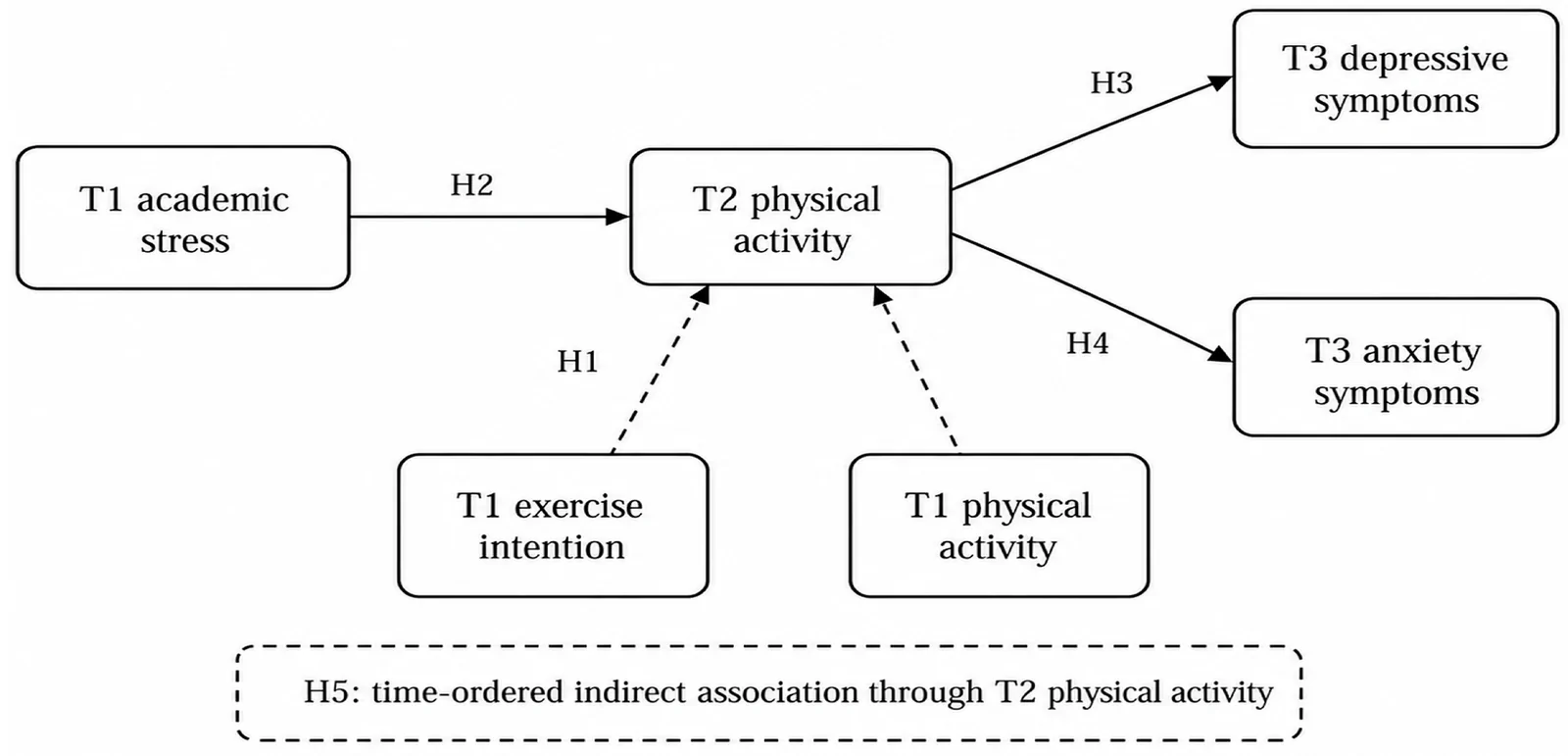
Revised conceptual model. All boxes represent directly measured variables. T2 physical activity is the primary intermediate variable. Dashed arrows from T1 exercise intention and T1 physical activity indicate prior predictors/covariates. H5 denotes the time-ordered indirect association through T2 physical activity; the residualized sensitivity parameterization is omitted from the figure as a separate construct.

All primary hypotheses were evaluated with the covariate set specified in Section 2.5. A secondary Stress × Intention analysis tested slope moderation, while residualized and quadratic specifications were treated as interpretive sensitivity analyses. The conceptual model is shown in Figure 1.

## 2. Materials and Methods

### 2.1. Design, Setting, and Ethics

This prospective observational study used three survey waves conducted at Zhengzhou University, a comprehensive university in Henan Province, China. T1 was administered in September 2025, T2 in December 2025, and T3 in March 2026, with approximately three months between waves. The timing allowed academic stress and baseline variables to precede T2 activity, and T2 activity to precede T3 symptoms; it also means that academic-calendar and seasonal influences cannot be fully separated. Reporting was organized with reference to the STROBE guidance for cohort studies (von Elm et al., 2007).

The study was conducted in accordance with the Declaration of Helsinki and was approved by the Ethics Committee of Zhengzhou University (protocol code ZZU-2025-87). All participants provided informed consent before participation. Anonymous identifiers were used for wave matching, and the analysis file did not include names, contact details, or direct identifiers.

### 2.2. Participants and Recruitment

Students were recruited through online class-coordinator networks spanning five broad academic areas: humanities, sciences, engineering, business, and medicine. This was convenience recruitment facilitated through classes rather than probability sampling. Eligible participants were enrolled students aged 18 years or older who could complete the questionnaire independently and consented to longitudinal follow-up. Responses were excluded from the longitudinal analytic sample when identifiers could not be matched across waves or when a focal variable was missing.

Fieldwork records documented 1078 invitations, 982 completed T1 surveys, 854 records matched at T2, and 750 records matched across all three waves. The final complete-case analytic file therefore represented 76.4% of T1 completers. It contained 400 men (53.3%) and 350 women (46.7%), with a mean age of 20.03 years (SD = 1.44; range 18–22) and a mean body mass index of 22.53 kg/m^2^ (SD = 3.03). To evaluate attrition, the 750 retained participants were compared with the 232 baseline participants not included in the three-wave analysis on all available T1 variables. No statistically significant differences were detected for age (test statistic = 0.254, *p* = 0.799), gender (0.670, *p* = 0.503), BMI (0.220, *p* = 0.826), academic stress (1.504, *p* = 0.133), exercise intention (0.664, *p* = 0.507), physical activity (−0.374, *p* = 0.708), PHQ-9 (−1.284, *p* = 0.200), or GAD-7 (0.124, *p* = 0.901). These comparisons provide no evidence of differential attrition on the observed baseline variables, but they do not establish missing completely at random or exclude selection on unmeasured characteristics. At T1, PHQ-9 and GAD-7 were completed in the same electronic questionnaire session and stored on the same row under a single Participant ID; no separate PHQ-9/GAD-7 merge was performed. Wave files were merged by strict one-to-one inner joins on this ID: 854 T2 matches (128 unmatched) and 750 three-wave matches (104 additional unmatched), with unmatched or incomplete records excluded.

### 2.3. Measures

Academic stress (T1): Perceived academic stress was assessed with the 18-item Perception of Academic Stress Scale, which addresses expectations, workload and examinations, and academic self-perceptions (Bedewy & Gabriel, 2015). Items are rated on a five-point scale; positively worded items were reverse-scored before summing the 18 items, yielding a theoretical range of 18–90. Item-level reconstruction in the *N* = 982 baseline file produced Cronbach’s α = 0.972. The observed composite in the analytic sample ranged from 18 to 88 (M = 56.08, SD = 10.50).

Exercise intention (T1): Exercise intention was assessed in September 2025 using three items adapted from Ajzen’s (2006) questionnaire-construction guidance: intention, planning, and effort to engage in regular exercise over the following three months. Items were rated on a continuous 1–7 visual analogue scale and averaged; higher scores indicated stronger intention (M = 5.44, SD = 1.03, observed range 1.73–7.00). The scale had Cronbach’s α = 0.88 in the original item-level export. The intention horizon and the T2 IPAQ-SF recall window were prospective but not fully time matched.

Physical activity (T1 and T2): Physical activity (PA) was assessed using the official Chinese version of the International Physical Activity Questionnaire—Short Form (IPAQ-SF; Craig et al., 2003), which has demonstrated acceptable test–retest reliability (ICC = 0.55) and moderate concurrent validity against accelerometer-measured activity in Chinese college student populations (Gao et al., 2022). Participants recalled their physical activities over the past 7 days, reporting the frequency (days) and duration (minutes) of three intensity categories: walking, moderate-intensity activities, and vigorous-intensity activities. The intention measure referred to the upcoming three months, whereas the T2 IPAQ-SF captured activity during the final seven days of that interval; the two measures therefore provided prospective but not fully time-matched assessments.

Weekly PA energy expenditure was calculated in Metabolic Equivalent task-minutes per week (MET-minutes/week) according to the official IPAQ scoring protocol (IPAQ Research Committee, 2005): Walking MET-minutes/week = 3.3 × minutes × days; Moderate PA MET-minutes/week = 4.0 × minutes × days; and Vigorous PA MET-minutes/week = 8.0 × minutes × days. The total PA score was the sum of these three sub-components. To ensure data quality and eliminate unrealistic responses, a two-stage data-cleaning protocol was applied. Following the IPAQ scoring protocol, any activity category exceeding 180 min per day (3 h) was truncated to 180 min to prevent single-day over-reporting from inflating sub-scores (IPAQ Research Committee, 2005). No participant in the matched analytic sample (*N* = 750) required additional exclusion. Self-reported IPAQ scores are nonetheless subject to recall and reporting error, and their agreement with device-measured activity is imperfect (Lee et al., 2011).

Depressive symptoms (T1 and T3): Depressive symptoms were measured with the nine-item Patient Health Questionnaire (PHQ-9), with items scored from 0 to 3 and summed to a 0–27 severity score (Kroenke et al., 2001). Evidence supports the reliability and validity of the Chinese version (Wang et al., 2014). Independent reconstruction from the *N* = 982 T1 item-level file yielded Cronbach’s α = 0.881. T1 PHQ-9 was included as a baseline covariate and T3 PHQ-9 was the depressive-symptom outcome.

Anxiety symptoms (T1 and T3): Anxiety symptoms were measured with the seven-item Generalized Anxiety Disorder scale (GAD-7), scored from 0 to 3 and summed to a 0–21 severity score (Spitzer et al., 2006). The scale has demonstrated acceptable psychometric properties among Chinese university students (C. Zhang et al., 2021). Independent reconstruction from the *N* = 982 T1 item-level file yielded Cronbach’s α = 0.845. T1 GAD-7 was included as a baseline covariate and T3 GAD-7 was the anxiety-symptom outcome.

Covariates: Every primary component-path, direct-effect, and total-effect model used the same covariate set: reported gender (0 = man, 1 = woman), age, BMI, T1 exercise intention, T1 physical activity, T1 PHQ-9, and T1 GAD-7. Using identical covariates permits exact decomposition of each total association into its direct and indirect components and avoids differences caused solely by model specification.

### 2.4. Conditional Modeling of T2 Physical Activity

Raw T2 physical activity was the primary intermediate variable. Because T1 exercise intention and T1 physical activity were included in all focal models, its association with academic stress and later symptoms was interpreted conditional on prior motivation and behavior. For comparison with the original formulation, we also calculated a sign-reversed residualized activity score by regressing T2 activity on T1 intention and T1 activity and multiplying the residual by −1.

For sensitivity analysis, the sign-reversed residualized T2 activity score was defined as

Residualized score_T2_ = Predicted PA_T2_ − PA_T2_

where Predicted PA_T2_ was obtained from an ordinary least-squares regression of T2 physical activity on T1 exercise intention and T1 physical activity in the locked *N* = 750 analytic dataset.

Positive residualized values indicate lower activity than predicted from T1 intention and T1 activity; they do not indicate that a participant failed to meet a personally specified exercise target. By ordinary least-squares construction, the residualized score had a mean of approximately zero and was orthogonal to T1 intention and T1 physical activity in the locked analytic sample.

The residualized score is a conditional linear re-expression of T2 physical activity rather than an independently observed psychological construct. In outcome regressions containing the same prior intention, prior activity, and covariates, the raw-activity and residualized parameterizations yield identical fitted values and R^2^, with coefficients of equal magnitude and opposite sign. Primary substantive conclusions are therefore stated in terms of raw T2 physical activity; the residualized score is retained only to demonstrate the equivalent conditional interpretation.

### 2.5. Statistical Analysis

Analyses were performed in Python 3.13.5 using pandas 2.2.3, NumPy 2.3.5, SciPy 1.17.0, and statsmodels 0.14.6. Data checks assessed duplicate identifiers, missing values, plausible ranges, and wave matching. Retained and attrited baseline participants were compared on all available T1 variables. The original *N* = 982 item-level baseline file was independently used to reconstruct PASS, PHQ-9, and GAD-7 scores and to verify coding and matching. Descriptive statistics, Pearson correlations, symptom-severity distributions, and paired changes were calculated. Two-sided tests used α = 0.05. The audit additionally verified the same-record T1 PHQ-9/GAD-7 structure, strict one-to-one ID linkage across waves, exact total-score reconstruction, the complete 9 × 7 PHQ-9 × GAD-7 cross-item Pearson correlation matrix, and identifier uniqueness before and after merging (Supplementary Table S1).

H1 was tested with an HC3-robust ordinary least-squares regression of T2 physical activity on T1 intention, T1 activity, T1 academic stress, gender, age, BMI, T1 PHQ-9, and T1 GAD-7. H2 used the same model and focused on the academic-stress coefficient. H3 and H4 regressed each T3 symptom outcome on raw T2 physical activity, T1 academic stress, and the identical covariate set. Standardized coefficients were reported where available, and variance-inflation factors assessed multicollinearity.

For H5, each total-effect, direct-effect, and component-path equation used the identical covariate set (Loh & Ren, 2023). Indirect associations were assessed with 5000 percentile bootstrap resamples. As a conservative generated-variable check, the residualization regression was refitted inside each resample; because the raw and residualized parameterizations are algebraically equivalent under identical adjustment, the indirect association has the same substantive interpretation. A non-significant direct coefficient was not interpreted as complete mediation.

**Figure 2:**
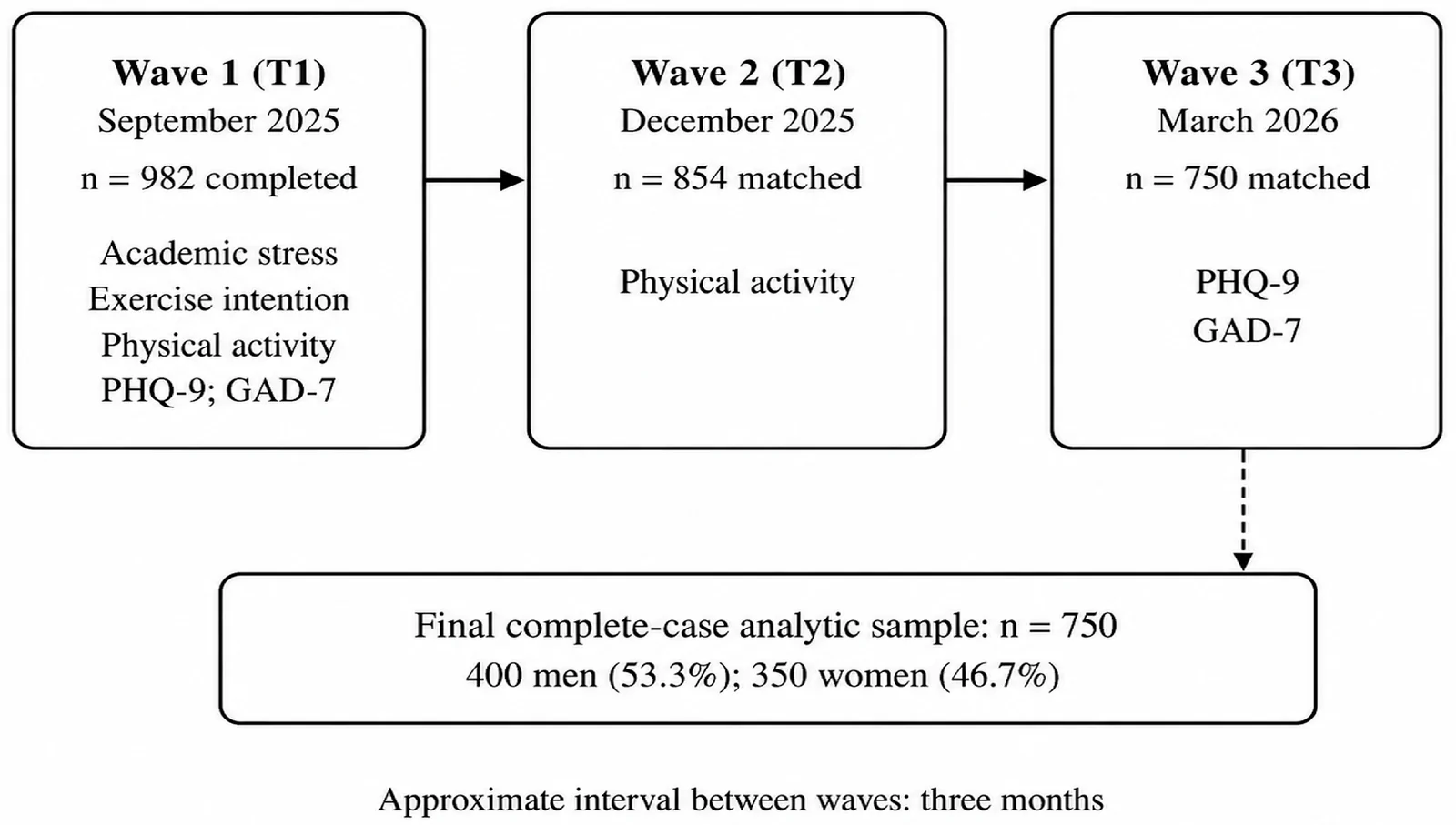
Three-wave study timeline and sample flow. The final analytic sample retained 750 of 982 baseline participants (76.4%). Raw T2 physical activity was the primary intermediate variable; residualized activity was examined only as an equivalent parameterization. Solid arrows indicate longitudinal progression and wave matching; the dashed arrow indicates derivation of the final complete-case analytic sample.

Secondary analyses added a centered Stress × Intention product term to test whether academic stress changed the prospective intention–activity slope. Parallel raw-activity and residualized outcome models were compared directly. For the quadratic sensitivity tests, the sign-reversed residualized activity score was divided by 100 before forming the squared term; the linear and squared terms were entered together with T1 academic stress, gender, age, BMI, T1 exercise intention, T1 physical activity, T1 PHQ-9, and T1 GAD-7. HC3 heteroscedasticity-consistent standard errors and 95% confidence intervals were reported. The three-wave study timeline and sample flow are shown in Figure 2.

## 3. Results

### 3.1. Data Integrity, Sample Characteristics, and Change over Time

The analytic file contained 750 unique participant identifiers, no missing values in the required variables, and no duplicate identifiers. The retention analysis detected no statistically significant baseline differences between the 750 retained and 232 attrited participants across age, gender, BMI, academic stress, exercise intention, physical activity, PHQ-9, or GAD-7 (all *p* = 0.133–0.901). These results reduce concern about selective attrition on the observed variables but do not prove that attrition was completely random. Mean physical activity decreased by 239.22 MET-minutes/week from T1 to T2 (95% CI [−276.07, −202.37], t(749) = −12.74, *p* < 0.001, d_z = −0.47). Mean PHQ-9 decreased by 1.43 points and mean GAD-7 by 1.11 points across the observed intervals; these group-level changes are distinct from the individual-difference associations tested in the regression models. The respondent-level audit confirmed the same-record baseline linkage and one-to-one longitudinal merge: 982 unique T1 IDs, 854 T2 matches, 750 three-wave matches, and no duplicate IDs before or after merging; unmatched records were excluded (Supplementary Table S1).

At T3, 46.3% of participants were in the PHQ-9 minimal range, 44.5% mild, 8.9% moderate, and 0.3% moderately severe; none had a score in the severe range. For GAD-7, 64.7% were in the minimal range, 32.7% mild, and 2.7% moderate; none had a score in the severe range. These categories are descriptive and the primary analyses retained the continuous scores. Descriptive statistics for the analytic sample are presented in Table 2.

The focal correlations are shown in Table 3. Academic stress correlated with lower T2 activity (r = −0.216), and T2 activity correlated inversely with T3 depressive symptoms (r = −0.261) and anxiety symptoms (r = −0.156). In the *N* = 750 analytic sample, T1 PHQ-9 and T1 GAD-7 were weakly and negatively correlated (r = −0.055). We therefore returned to the original *N* = 982 item-level baseline file and performed a respondent-level and item-level audit. PHQ-9 and GAD-7 were collected in the same T1 electronic session and stored on the same row under the same Participant ID; no separate PHQ-9/GAD-7 merge occurred. All 982 reconstructed totals matched the stored totals exactly. Mean within-scale item correlations were 0.451 for PHQ-9 and 0.437 for GAD-7, while the 63 cross-scale item correlations averaged −0.027 (range −0.090 to 0.036; complete 9 × 7 matrix in Supplementary Table S1). The reconstructed baseline scale-total correlation remained small and non-significant (r = −0.053, *p* = 0.100). Together with one-to-one longitudinal ID matching, these checks found no evidence of scoring or participant-level alignment error, although the near-zero baseline association remains an unusual sample-specific limitation.

### 3.2. Exercise Intention and Subsequent Physical Activity (H1)

H1 was supported. In the fully adjusted T2 activity model, stronger T1 exercise intention was associated with higher T2 physical activity (B = 155.963, SE = 13.593, *β* = 0.308, 95% CI [129.322, 182.605], *p* < 0.001). T1 physical activity was also strongly associated with T2 activity (*β* = 0.617), whereas academic stress was associated with lower T2 activity (B = −12.771, SE = 1.313, *β* = −0.260, *p* < 0.001). The model explained 52.5% of T2 activity variance. Variance-inflation factors were below 1.16, indicating that multicollinearity did not explain the results. Full regression results are presented in Table 4.

### 3.3. Academic Stress and Subsequent Physical Activity (H2)

H2 was supported. In the fully adjusted T2 activity model, higher T1 academic stress was associated with lower T2 physical activity (B = −12.771, SE = 1.313, *β* = −0.260, 95% CI [−15.344, −10.198], *p* < 0.001). Thus, at the same T1 intention, T1 activity, demographic characteristics, and baseline symptom levels, a one-point higher stress score corresponded to approximately 12.8 fewer MET-minutes/week at T2. The Stress × Intention test reported below was not significant, so this is interpreted as an additive conditional association rather than evidence that stress weakened the intention–activity slope.

### 3.4. Subsequent Physical Activity and Later Symptoms (H3–H5)

H3 and H4 were supported in the verified raw-activity models. Under identical adjustment for T1 academic stress, gender, age, BMI, T1 exercise intention, T1 physical activity, T1 PHQ-9, and T1 GAD-7, higher T2 physical activity was associated with lower T3 depressive symptoms (B = −0.0024, SE = 0.0002, 95% CI [−0.0029, −0.0020], *p* < 0.001; model R^2^ = 0.4257) and lower T3 anxiety symptoms (B = −0.0013, SE = 0.0002, 95% CI [−0.0017, −0.0008], *p* < 0.001; model R^2^ = 0.2720). The sign-reversed residualized coefficients were equal in magnitude and opposite in sign, and the fitted values and R^2^ were identical. The residualized score therefore supplies no additional linear predictive information beyond raw T2 activity under the same specification. These parallel model results are summarized in Table 5.

**Figure 3:**
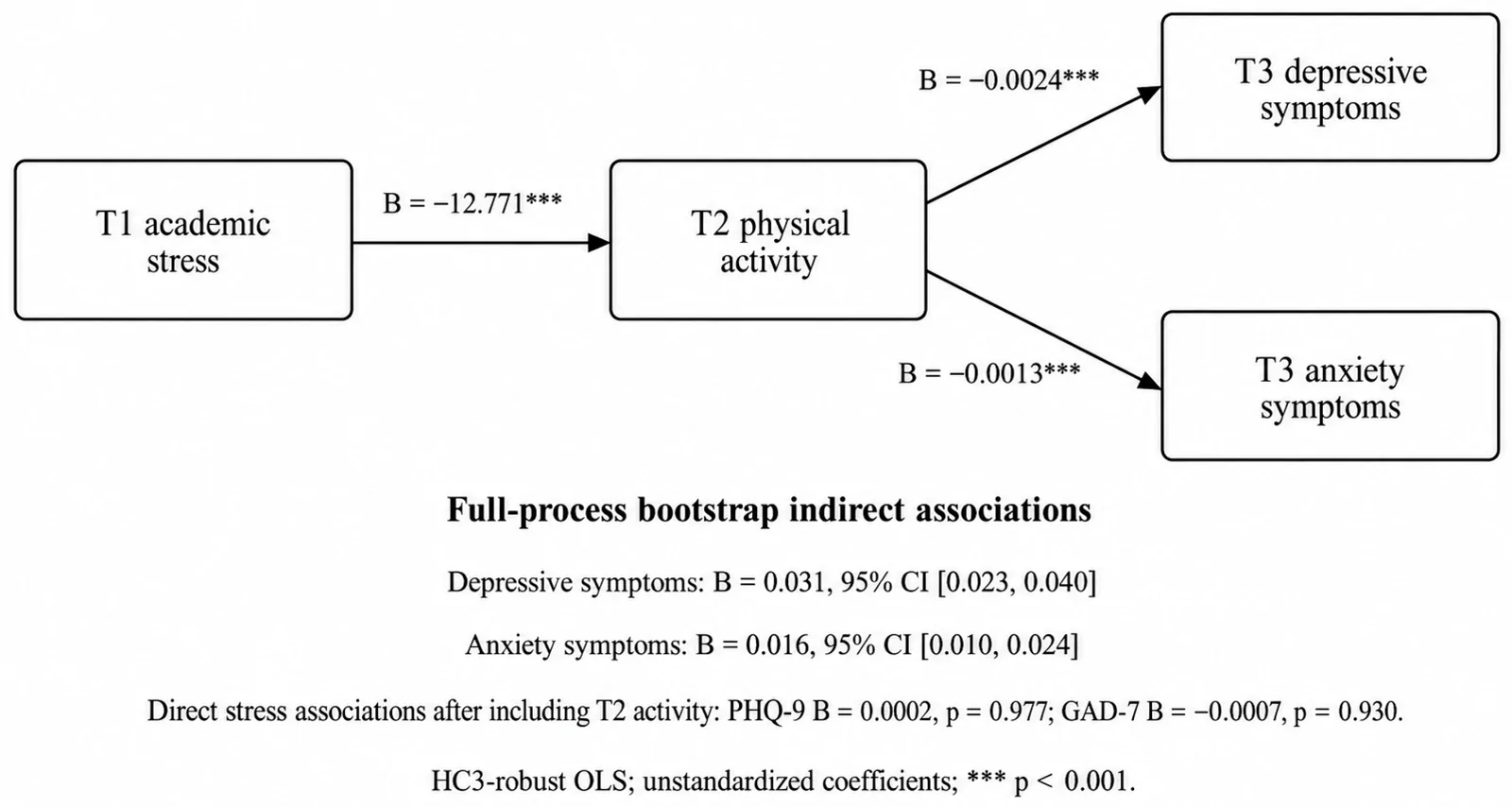
Revised empirical longitudinal model using raw T2 physical activity as the primary intermediate variable. Unstandardized coefficients are shown; *** *p* < 0.001. Indirect confidence intervals were obtained from the 5000-resample full-process bootstrap; the displayed component, direct, and indirect estimates correspond to Table 6.

H5 was supported as a time-ordered statistical indirect association. For depressive symptoms, the component paths were a = −12.7707 and b = −0.002423; the total association was c = 0.0312 (SE = 0.0086, 95% CI [0.0143, 0.0481], *p* < 0.001), the direct association was c′ = 0.0002 (SE = 0.0084, 95% CI [−0.0161, 0.0166], *p* = 0.977), and the bootstrap indirect association was ab = 0.0309 (bootstrap SE = 0.0042, 95% CI [0.0229, 0.0396]). For anxiety symptoms, a = −12.7707 and b = −0.001272; c = 0.0155 (SE = 0.0082, 95% CI [−0.0006, 0.0316], *p* = 0.059), c′ = −0.0007 (SE = 0.0081, 95% CI [−0.0167, 0.0153], *p* = 0.930), and ab = 0.0162 (bootstrap SE = 0.0035, 95% CI [0.0099, 0.0236]). In each outcome, c = c′ + ab exactly using the unrounded estimates; minor discrepancies from multiplication of displayed coefficients reflect rounding. These quantities are model-based decompositions within observational data, not causal mediation effects. The full decomposition is reported in Table 6, and the corresponding empirical longitudinal model is shown in Figure 3.

### 3.5. Secondary and Sensitivity Analyses

The centered Stress × Intention interaction did not predict T2 physical activity (B = −1.341, SE = 1.341, 95% CI [−3.969, 1.288], *p* = 0.317). Thus, the data did not provide evidence that academic stress changed the prospective intention–activity slope. The supported result is an additive association between higher stress and lower later activity at a given level of prior intention and activity.

Parallel raw-activity and sign-reversed residualized models confirmed exact algebraic equivalence. For PHQ-9, the coefficients were −0.0024 and 0.0024 and both models had R^2^ = 0.4257. For GAD-7, the coefficients were −0.0013 and 0.0013 and both models had R^2^ = 0.2720. The residualized parameterization therefore changes only the sign and conditional framing, not predictive information or model fit.

Formal quadratic tests under the final verified specification did not indicate curvature. The quadratic term was not significant for PHQ-9 (B = 0.0052, SE = 0.0038, 95% CI [−0.0022, 0.0127], *p* = 0.168) or GAD-7 (B = 0.0033, SE = 0.0048, 95% CI [−0.0060, 0.0126], *p* = 0.491). Accordingly, the previous piecewise/asymmetry interpretation and the corresponding figure remain removed, and H3–H5 are interpreted as average linear associations across the observed activity range.

Together, the interaction, equivalent-parameterization, and quadratic checks support a cautious interpretation centered on conditional subsequent physical activity rather than on a distinct intention-failure construct. A summary of these secondary and sensitivity analyses is provided in Table 7.

## 4. Discussion

### 4.1. Principal Findings

Higher academic stress was associated with lower subsequent physical activity, which was in turn associated with higher later depressive and anxiety symptoms. Stronger T1 exercise intention was also associated with higher T2 activity. With identical covariates across component, direct, and total-effect equations, full-process bootstrap intervals supported time-ordered indirect associations from T1 academic stress to both T3 symptom outcomes through T2 physical activity.

Three qualifications define the interpretation. First, the Stress × Intention interaction was not significant, so the study does not show that academic stress weakened the intention–activity slope. Second, raw T2 activity and the residualized parameterization were algebraically equivalent under identical controls; the residual does not provide incremental validity or identify a separate psychological mechanism. Third, the formal quadratic terms were not significant, so the revised results do not support a claim that the association is confined to one side of the residual distribution.

### 4.2. Academic Stress and Conditional Physical Activity

The negative association between academic stress and subsequent physical activity is consistent with the possibility that academic demands compete with health behavior. Students facing workload, examinations, and performance concerns may have less discretionary time and greater fatigue or rumination (Barbayannis et al., 2022; Bedewy & Gabriel, 2015; Campbell et al., 2022). Post-intentional theories identify planning, barrier management, self-monitoring, and goal shielding as possible determinants of activity after intention has formed (Feil et al., 2023; Rhodes & de Bruijn, 2013; Schwarzer, 2008; Sniehotta et al., 2005). These processes were not measured, so they are possible explanations rather than demonstrated mediators.

The non-significant interaction prevents the more specific conclusion that stress altered the strength of the intention–activity relationship. An additive coefficient indicates lower activity at higher stress after conditioning on intention and prior activity; a moderation coefficient would indicate that intention predicts activity less strongly at high stress. Only the additive pattern was supported. Future work should measure intention and behavior over the same short interval and repeatedly assess planning, habit, perceived control, and schedule instability.

### 4.3. Subsequent Physical Activity and Later Symptoms

Higher T2 physical activity was associated with lower depressive and anxiety symptoms at T3 beyond both baseline symptom scores. This association may reflect opportunities for mood regulation, mastery, social interaction, sleep support, and recovery that accompany activity (Bull et al., 2020; McDowell et al., 2019; Pearce et al., 2022). A discrepancy interpretation is also conceivable, but participants did not report a target activity amount, remembered goal attainment, frustration, competence, or perceived failure. The data therefore support a conditional activity association, not a demonstrated self-discrepancy mechanism.

The revised formal nonlinearity tests did not support curvature for either outcome. The single linear coefficient is therefore retained as an average association across the observed range, while recognizing that no observational model can rule out more complex functional forms in other samples. Because the residualized and raw-activity models are algebraically equivalent, the absence of quadratic evidence also removes the basis for interpreting positive residuals as a distinct psychological subgroup.

The total stress association was statistically significant for depressive symptoms but narrowly non-significant for anxiety symptoms (*p* = 0.059). A significant indirect association does not require a significant total association, because direct and indirect components may differ in magnitude or direction. The difference between outcomes should not be interpreted as proof that the activity pathway is depression-specific, because equality of the two total associations was not directly tested. Sleep, social support, financial pressure, academic performance, personality, prior clinical history, and general self-regulation may also contribute to both activity and symptoms.

### 4.4. Mean Change, Academic Calendar, and Seasonality

Physical activity, depressive symptoms, and anxiety symptoms all declined on average across the observed intervals. Group-level mean trends and individual-difference associations answer different questions: students with lower conditional T2 activity had relatively higher later symptoms, but the cohort-level activity decline did not cause the cohort-level symptom trend. September, December, and March also differ in weather, daylight, course demands, examinations, holidays, and academic-term transitions. These contextual influences cannot be separated without repeated T2 stress and symptom assessments.

### 4.5. Methodological Role of the Residualized Parameterization

The analysis illustrates the limited but transparent role of residualization. The primary substantive variable is raw T2 physical activity conditional on T1 intention and T1 activity. A sign-reversed residual can display the same coefficient as activity below a model-predicted level, but it is sample- and model-dependent and contains no additional linear information when its component predictors are included. It should therefore not be described as a new construct, a participant-specific goal deficit, or evidence of intention failure.

A stronger intention–behavior design would define intention and behavior for the same target, action, context, unit, and period. Participants might report planned minutes of moderate-to-vigorous leisure-time exercise for the next seven days, followed by device-measured or diary-recorded minutes during that same week. Repeated weekly measures would distinguish changes in intention from failures of enactment and permit direct assessment of perceived discrepancy beyond activity volume.

### 4.6. Practical Implications

The findings suggest that campus programs should consider barriers associated with academic stress and reduced activity, but the study did not test an intervention. Short activity bouts, nearby opportunities, peer-supported routines, and flexible alternatives during examinations may reduce the practical cost of remaining active (Bélanger-Gravel et al., 2013; Gollwitzer, 1999; Sniehotta et al., 2005). These are hypotheses for intervention research rather than recommendations demonstrated by the present data.

Programs should avoid framing lower activity as personal failure. Students may be adapting to constrained time and resources, and supportive approaches should reduce friction and integrate activity options with broader stress-management and mental-health services (Amanvermez et al., 2023). Experimental trials are required to establish whether such approaches alter activity or symptoms.

### 4.7. Strengths and Limitations

Several strengths should be noted. The three-wave design separated stress, subsequent activity, and later symptoms; all primary total, direct, and component-path models used identical covariates; generated-variable uncertainty was addressed through a full-process bootstrap; raw and residualized parameterizations were compared explicitly; attrition was examined across eight observed baseline variables; and item-level baseline scores were reconstructed to audit the unusual PHQ-9/GAD-7 correlation. The respondent-level and item-level data audit is reported in Supplementary Table S1.

Several limitations warrant consideration. First, the observational design cannot establish causal direction. T2 symptoms and T2 academic stress were not measured, so emerging symptoms or changing stress between T1 and T2 may have contributed to T2 activity. Second, all focal variables were self-reported, and IPAQ-SF activity is vulnerable to recall and social-desirability error (Podsakoff et al., 2012). Third, intention referred to exercise over three months, whereas T2 activity covered the preceding seven days and included non-exercise activity; the study therefore did not directly measure an intention–behavior gap.

Fourth, the residualized parameterization is model-dependent and algebraically equivalent to raw T2 activity under identical covariates. Fifth, 76.4% of baseline participants were retained. No observed T1 variable differed significantly between retained and attrited groups, but these comparisons do not prove missing completely at random and cannot rule out attrition related to unmeasured variables. Sixth, class or department identifiers were unavailable, so HC3 standard errors could not address within-class clustering.

Seventh, the respondent-level and item-level audit confirmed exact agreement between stored and reconstructed T1 PHQ-9 and GAD-7 totals for all 982 baseline records, with no duplicate identifiers detected before or after wave merging. At T1, both scales were stored in the same questionnaire record under the same Participant ID, and longitudinal wave files were matched one-to-one; unmatched records were excluded. Mean within-scale item correlations were positive (PHQ-9: r = 0.451; GAD-7: r = 0.437), whereas the mean cross-scale item correlation was −0.027; the complete 9 × 7 cross-item matrix is provided in Supplementary Table S1. Nevertheless, the near-zero baseline PHQ-9/GAD-7 scale-total correlation remained (r = −0.053, *p* = 0.100). This unusual sample-specific pattern should therefore still be interpreted cautiously despite the absence of identified scoring or participant-alignment errors. The T3 item-level export was not included in the de-identified rerun package, so T3 wave-specific reliability was not independently recalculated in this revision. Eighth, the sample came from one university and may not generalize to other institutions, regions, or educational systems. Ninth, unmeasured factors—including sleep, injuries, workload, social support, financial strain, and prior clinical history—may confound the reported associations.

### 4.8. Directions for Future Research

Future studies should combine device-measured activity with time-matched intention measures, obtain stress and symptom assessments at every wave, record perceived goal attainment, and use multi-site recruitment with cluster-aware inference. More frequent assessment would support within-person models and distinguish stable between-person differences from short-term deviations (Hamaker et al., 2015). Preregistered trials are needed before planning or low-friction activity support can be presented as a mechanism for improving mental health.

## 5. Conclusions

Among 750 of 982 baseline participants followed across three waves (retention = 76.4%), higher academic stress was associated with lower subsequent physical activity after adjustment for prior exercise intention, prior activity, demographics, and both baseline symptom measures. Lower T2 activity was in turn statistically associated with later depressive and anxiety symptoms, and full-process bootstrap analyses supported time-ordered indirect associations. The data did not show that stress weakened the intention–activity slope, did not support quadratic nonlinearity in the revised models, and did not show that a residualized score adds information beyond raw activity. Taken together, the findings support a longitudinal association among academic stress, relatively lower subsequent physical activity, and later symptoms; they do not establish causality or subjective intention failure.

## Figures and Tables

**Table 1:** Summary of hypotheses, analyses, key results, and defensible interpretations.

Hypothesis	Analysis	Key Result	Defensible Interpretation
H1	HC3 OLS: T1 intention → T2 PA	*β* = 0.308, *p* < 0.001	Stronger prior intention was associated with higher subsequent physical activity.
H2	HC3 OLS: T1 stress → T2 PA	B = −12.771, *β* = −0.260, *p* < 0.001	Higher academic stress was associated with lower subsequent physical activity after covariate adjustment.
H3	HC3 OLS: T2 PA → T3 PHQ-9	B = −0.0024, *p* < 0.001	Higher T2 physical activity was associated with lower later depressive symptoms.
H4	HC3 OLS: T2 PA → T3 GAD-7	B = −0.0013, *p* < 0.001	Higher T2 physical activity was associated with lower later anxiety symptoms.
H5	5000-resample full-process bootstrap	PHQ-9: B = 0.0309; GAD-7: B = 0.0162; both bootstrap CIs excluded 0	T2 physical activity statistically accounted for time-ordered indirect associations; this is not evidence of causal mediation.

Note. PA = physical activity. All primary models adjusted for gender, age, BMI, T1 exercise intention, T1 physical activity, T1 PHQ-9, and T1 GAD-7. The residualized activity score was examined only as an algebraically equivalent sensitivity parameterization.

**Table 2:** Descriptive statistics for the analytic sample (*N* = 750).

Variable	M	SD	Minimum	Maximum
Age (years)	20.03	1.44	18.00	22.00
BMI (kg/m^2^)	22.53	3.03	14.40	32.40
Academic stress at T1	56.08	10.50	18.00	88.00
Exercise intention at T1	5.44	1.03	1.73	7.00
Physical activity at T1 (MET-min/week)	1527.36	614.20	0.00	3501.00
Physical activity at T2 (MET-min/week)	1288.13	520.35	0.00	3190.00
Sign-reversed residualized T2 PA (secondary)	0.00	381.13	−1615.87	1095.64
PHQ-9 at T1	6.42	3.80	0.00	21.00
PHQ-9 at T3	4.99	3.19	0.00	16.00
GAD-7 at T1	4.77	3.01	0.00	16.00
GAD-7 at T3	3.66	2.74	0.00	13.00

Note. BMI = body mass index; PHQ-9 = Patient Health Questionnaire-9; GAD-7 = Generalized Anxiety Disorder-7; PA = physical activity. The residualized activity score is centered near zero by construction and is not treated as a distinct psychological construct.

**Table 3:** Pearson correlations among focal variables.

Variable	1	2	3	4	5	6	7	8	9
1. Academic stress T1	1								
2. Exercise intention T1	−0.004	1							
3. PA T1	0.079 *	0.002	1						
4. PA T2	−0.216 ***	0.308 ***	0.598 ***	1					
5. Reversed residual T2	0.354 ***	−0.000	−0.000	−0.740 ***	1				
6. PHQ-9 T1	0.022	0.005	0.001	−0.011	0.018	1			
7. GAD-7 T1	0.034	0.050	−0.001	−0.034	0.065	−0.055	1		
8. PHQ-9 T3	0.124 ***	0.020	−0.043	−0.261 ***	0.326 ***	0.567 ***	−0.000	1	
9. GAD-7 T3	0.078 *	0.007	0.004	−0.156 ***	0.217 ***	−0.121 ***	0.483 ***	0.317 ***	1

Note. * *p* < 0.05, *** *p* < 0.001. The reversed residual is orthogonal to T1 exercise intention and T1 PA by construction; it is shown only as an equivalent parameterization of T2 PA.

**Table 4:** HC3-robust regression predicting T2 physical activity.

Predictor	B	SE	*β*	95% CI for B	*p*	Result
Exercise intention T1	155.963	13.593	0.308	[129.322, 182.605]	<0.001	Significant
Physical activity T1	0.523	0.022	0.617	[0.480, 0.566]	<0.001	Significant
Academic stress T1	−12.771	1.313	−0.260	[−15.344, −10.198]	<0.001	Significant
Gender (0 man, 1 woman)	−34.431	26.667	−0.033	[−86.698, 17.836]	=0.197	Not significant
Age	−3.865	9.043	−0.011	[−21.589, 13.858]	=0.669	Not significant
BMI	−5.454	4.524	−0.031	[−14.320, 3.413]	=0.228	Not significant
PHQ-9 T1	−1.294	3.674	−0.009	[−8.495, 5.907]	=0.725	Not significant
GAD-7 T1	−7.264	4.680	−0.040	[−16.437, 1.909]	=0.121	Not significant

Note. *N* = 750. Model R^2^ = 0.525; adjusted R^2^ = 0.520. HC3 heteroscedasticity-consistent standard errors were used.

**Table 5:** Parallel raw-activity and residualized outcome models under identical covariate adjustment.

Outcome	Raw PA B	Residualized B	*p*	Raw PA R^2^	Residual R^2^	Interpretation
PHQ-9 at T3	−0.0024	0.0024	<0.001	0.4257	0.4257	Same fit; opposite signs
GAD-7 at T3	−0.0013	0.0013	<0.001	0.2720	0.2720	Same fit; opposite signs

Note. All models used the identical covariate set. The residualized score was sign reversed, so its coefficient has the opposite sign. Equal R^2^ values demonstrate that the two parameterizations contain the same linear predictive information.

**Table 6:** Component, direct, total, and full-process bootstrap indirect associations using identical covariates.

Outcome	Path	B	SE	*p*	95% CI	Identity
PHQ-9 T3	a: Stress T1 → raw PA T2	−12.7707	1.3129	<0.001	[−15.3439, −10.1975]	—
PHQ-9 T3	b: raw PA T2 → PHQ-9 T3	−0.002423	0.000223	<0.001	[−0.002861, −0.001985]	—
PHQ-9 T3	ab: indirect association	0.03095	0.0042	—	[0.0229, 0.0396]	—
PHQ-9 T3	c′: direct association	0.00024	0.0084	0.977	[−0.0161, 0.0166]	—
PHQ-9 T3	c: total association	0.03119	0.0086	<0.001	[0.0143, 0.0481]	c = c′ + ab
GAD-7 T3	a: Stress T1 → raw PA T2	−12.7707	1.3129	<0.001	[−15.3439, −10.1975]	—
GAD-7 T3	b: raw PA T2 → GAD-7 T3	−0.001272	0.000235	<0.001	[−0.001733, −0.000810]	—
GAD-7 T3	ab: indirect association	0.01624	0.0035	—	[0.0099, 0.0236]	—
GAD-7 T3	c′: direct association	−0.00072	0.0081	0.930	[−0.0167, 0.0153]	—
GAD-7 T3	c: total association	0.01552	0.0082	0.059	[−0.0006, 0.0316]	c = c′ + ab

Note. a = academic stress T1 → raw PA T2; b = raw PA T2 → T3 outcome; c = total association; c′ = direct association after including T2 activity; ab = indirect association. All equations adjusted for gender, age, BMI, T1 exercise intention, T1 physical activity, T1 PHQ-9, and T1 GAD-7. HC3 standard errors and confidence intervals are shown for a, b, c′, and c; the standard error and percentile confidence interval for ab are based on 5000 full-process bootstrap resamples. The residualized sensitivity parameterization was recomputed within each resample. In both outcome models, c = c′ + ab. Values for c′, ab, and c are reported to five decimal places to permit direct verification of c = c′ + ab; the very small b-path coefficients and their uncertainty estimates are retained to six decimal places to permit verification of the product term.

**Table 7:** Summary of secondary and sensitivity analyses.

Analysis	Outcome	Estimate 1	Estimate 2	Model Fit	*p*	Interpretation
Stress × intention	T2 PA	B = −1.341	SE = 1.341	—	0.317	No moderation
Raw vs. residual	PHQ-9 T3	Raw B = −0.0024	Residual B = 0.0024	R^2^ = 0.4257	<0.001	Identical fit
Raw vs. residual	GAD-7 T3	Raw B = −0.0013	Residual B = 0.0013	R^2^ = 0.2720	<0.001	Identical fit
Quadratic term test	PHQ-9 T3	B = 0.0052	SE = 0.0038	95% CI [−0.0022, 0.0127]	0.168	No curvature
Quadratic term test	GAD-7 T3	B = 0.0033	SE = 0.0048	95% CI [−0.0060, 0.0126]	0.491	No curvature

Note. The raw-activity and residualized models used identical covariates. Quadratic terms were estimated after dividing the residualized activity score by 100, jointly entering its linear and squared terms and the unified covariate set, and using HC3 standard errors. B and SE for the quadratic terms are expressed for the squared rescaled score. CI = confidence interval.

## Data Availability

The datasets presented in this article are not readily available because of privacy and ethical restrictions regarding the participants’ sensitive information. Requests to access the datasets should be directed to the corresponding author.

## Supplementary Materials

The following supporting information can be downloaded at: https://www.mdpi.com/article/10.3390/bs16091629/s1, Table S1: Participant-level ID linkage audit and complete 9 × 7 PHQ-9 × GAD-7 cross-item correlation matrix at baseline.
