# Supplementary material for: Academic Stress, Subsequent Physical Activity, and Depressive and Anxiety Symptoms Among College Students: A Three-Wave Longitudinal Study
Source: Behav Sci (Basel). 2026 Sep 11;16(9):1629. doi: 10.3390/bs16091629 (PMC13603287; doi:10.3390/bs16091629)
Supplement: Supplementary file 1 [file behavsci-16-01629-s001.zip › behavsci-4499525-supplementary.pdf]

Supplementary Information

Manuscript: Academic Stress, Subsequent Physical Activity, and Depressive and Anxiety Symptoms among College Students: A Three-Wave Longitudinal Study

Table S1. Participant-level linkage, longitudinal ID matching, and baseline PHQ-9 × GAD-7 item-level audit

A. Participant-level linkage and longitudinal merge audit

Audit item	Result	Validation / interpretation
T1 survey architecture	PHQ-9 and GAD-7 administered in the same electronic questionnaire battery during the same baseline survey session.	No separate PHQ/GAD file linkage was required.
T1 storage relationship	All baseline measures stored under one unique anonymous Participant ID in a single row of the original system export.	PHQ-9 and GAD-7 items belonged to the same submitted participant record.
Invitations issued at T1	1,078	Fieldwork invitations.
Completed T1 surveys	982	Baseline completers.
Unique T1 Participant IDs	982	All T1 IDs unique.
Duplicate IDs at T1	0	No duplicate baseline IDs.
Stored vs reconstructed PHQ-9 totals	982 / 982 exact agreement	No scoring discrepancy identified.
Stored vs reconstructed GAD-7 totals	982 / 982 exact agreement	No scoring discrepancy identified.
T2 records matched to T1	854	Exact Participant-ID match.
T1 → T2 unmatched / lost	128	Excluded from later complete-case analysis.
Three-wave matched records at T3	750	Complete T1–T2–T3 records.
T2 → T3 unmatched / lost	104	Excluded from final complete-case analysis.
Final complete-case sample	750 (76.4% of T1 completers)	Used for primary longitudinal analyses.
Longitudinal merge method	Exact one-to-one inner join on Participant ID	No fuzzy, approximate, or many-to-many matching.
Duplicate IDs after merging	0	No duplicate Participant IDs in the final analytic file.
Handling of unmatched / incomplete records	Excluded	No imputation or approximate ID recovery.

Note. At baseline, PHQ-9 and GAD-7 responses were recorded within the same submitted survey record under the same Participant ID. Longitudinal wave matching was performed only by exact Participant-ID matching.

B. Full baseline PHQ-9 × GAD-7 cross-item Pearson correlation matrix (N = 982)

PHQ-9 item	GAD-1 Nervous/anxious	GAD-2 Control worry	GAD-3 Excess worry	GAD-4 Relaxation	GAD-5 Restlessness	GAD-6 Irritability	GAD-7 Fear
PHQ-1 Anhedonia	−0.008	−0.050	−0.063*	−0.072*	−0.003	−0.045	0.014
PHQ-2 Low mood	−0.011	−0.047	−0.082*	−0.052	−0.013	−0.060	−0.007
PHQ-3 Sleep	−0.046	−0.090**	−0.056	−0.075*	−0.044	−0.042	0.009
PHQ-4 Fatigue	0.031	−0.012	0.021	−0.021	−0.019	−0.005	0.034
PHQ-5 Appetite	0.012	−0.025	−0.041	−0.017	−0.003	−0.032	0.034
PHQ-6 Negative self-view	−0.028	−0.055	−0.044	−0.057	−0.004	−0.037	0.036
PHQ-7 Concentration	−0.017	−0.061	−0.026	−0.021	−0.001	−0.024	0.025
PHQ-8 Psychomotor	−0.045	−0.049	−0.018	−0.064*	−0.062	−0.084**	0.001
PHQ-9 Self-harm thoughts	−0.008	−0.055	−0.010	−0.063*	−0.045	−0.015	0.002

*Note. * $p < 0.05$, ** $p < 0.01$, *** $p < 0.001$; two-tailed Pearson correlations.*

C. Correlation-structure summary

Summary statistic	Estimate	Additional information
PHQ-9 within-scale mean item correlation	r = 0.451	Range 0.415 to 0.505; Cronbach's α = 0.881
GAD-7 within-scale mean item correlation	r = 0.437	Range 0.377 to 0.478; Cronbach's α = 0.845
PHQ-9 × GAD-7 cross-scale mean item correlation	r = −0.027	Range −0.090 to 0.036 across 63 item pairs
Baseline PHQ-9 × GAD-7 scale-total correlation	r = −0.053, p = 0.100	Near-zero association retained as an unusual sample-specific limitation

Interpretive note. The complete cross-item matrix is reported as a transparency check. Correct baseline participant linkage is supported primarily by the same-record survey architecture: the PHQ-9 and GAD-7 were collected within the same baseline questionnaire submission under the same Participant ID and were not merged from separate symptom files. The near-zero baseline association remains unusual and should be interpreted cautiously.
